# Drug resistance and influencing factors of biofilm bacteria in upper urinary calculi patients with double J stent indwelling

**DOI:** 10.1186/s12894-023-01339-x

**Published:** 2023-10-14

**Authors:** Qian Chen, JunBing Ye, Xiao Bin Li, Ke Zeng, Shiping Zeng

**Affiliations:** 1 Department of Nephrology, First People’s Hospital of Zigong City, Zigong, China; 2 Department of Urology, First People’s Hospital of Zigong City, Zigong, China

**Keywords:** Biofilm bacteria, Drug resistance, Upper urinary calculi, Double J stent

## Abstract

**Objective:**

To analyze the distribution and drug resistance of biofilm bacteria infected with upper urinary calculi patients with double J stent indwelling, and to explore the influencing factors of Biofilm Bacteria Infections.

**Methods:**

A total of 400 patients with upper urinary calculi who adopted double J stent inserting in our hospital from January 2019 to January 2022 were included. Urine and double J stent samples were collected, pathogen cultures were performed, and then drug sensitivity test analysis was carried out for isolates. Univariate and multivariate logistic regression analyzes were used to analyze the influencing factors of patients with double J stent associated biofilm bacteria infections.

**Results:**

A total of 13 strains (3.2%) of biofilm bacteria were detected in urine samples and 168 strains (42%) in double J stent samples (P < 0.05), 95 strains (23.7%) of pathogenic bacteria were separated from urine samples and 117 strains (29.2%) from double J-stent samples (P > 0.05). *Escherichia coli* were the most common bacteria. There was significantly higher drug resistance observed in biofilm bacteria versus urine-cultured pathogens (P < 0.05). Advanced age, long-term catheterization, inadequate water intake, hypoproteinemia, abnormal renal function, and diabetes mellitus were independent risk factors for biofilm bacteria infection associated with double J stent(P < 0.05).

**Conclusion:**

Among the upper urinary calculi patients with double J stent indwelling, the positive rate and drug resistance of biofilm bacteria obtained from double J stent were significantly higher than that from urine. More attention should be paid to the factors that influence biofilm bacteria infections.

## Introduction

Urolithiasis is one of the common and multiple diseases of the urinary system with clinical manifestations of vomiting, low back pain, and hematuria, which seriously affects human health. The incidence of urolithiasis ranges from 7 to 13% in North America, 5 to 9% in Europe, 1 to 5% in Asia [1], and in China, that is 5.8% in adults [2]. With the vigorous development of intracavitary urology, double J stents have been extensively applied to the treatment of upper urinary calculi, ureteral injury, ureteral obstruction, or stenosis for the functions of relieving urinary obstruction, draining urine, and protecting renal function [3]. With the extension indwelling time, urinary tract infections associated with double J stent would inevitably occur and become a common complication. Without the normal protective mechanisms of body tissue, the surface of various implants could easily adhered by floating bacteria under the adhesion of bacterial flagella. After a period of adaptation, bacteria colonized rapidly and glycocalyx was produced, then plaque formed and exopolysaccharide mucus was secreted under the protection of the glycocalyx. Finally, the stable three-dimensional structure of the bacterial biofilm on the surface of the material gradually formed [4].This is a defense response of bacteria to adapt to the external environment and leads to the aggravation of drug resistance through a variety of mechanisms [5]. Therefore, we aim to analyze the distribution and drug resistance of pathogens infected with upper urinary calculi patients with double J stent indwelling and its influencing factors, and to provide references for the clinical treatment of biofilm bacteria infections.

## Materials and methods

### Source of data

A total of 400 patients with upper urinary calculi who adopted double J stent inserting in our urology unit from January 2019 to January 2022 were selected. The study was approved by the hospital ethics committee and written informed consent was obtained from the study participants. Inclusion criteria:①adults over 18 years old;②patients whose clinical manifestations and imaging examination met the diagnostic criteria for upper urinary calculi;③the 5F~6F double J stent(provided by the COOK company, USA) was inserted to relieve hydronephrosis, drain stone residue and dilate the ureter after laser lithotripsy for the last 7 days;④all patients were in good mental condition and were able to cooperate in completing this survey;⑤patients with complete clinical data. Exclusion criteria:①patients with urinary tract infection or obvious systemic infection before double J stent was inserted;②patients with contraindications to double J stent placement(such as severe hematuria, lumen obstruction, lower urinary tract symptoms, suprapubic pain, and low back pain, etc.);③patients who failed to get a double J stent inserted;④patients with postoperative infection from other causes or with obvious manifestations of systemic infection;⑤patients with incomplete clinical data;⑥paients with severe neurological or mental disease.⑦patients received antibacterial treatment during the placement of double J stent.

### Content of data

During the patient’s hospital stay, baseline data of patients were collected such as gender, age, diameter of stone(derived from CT examination), catheter indwelling time, daily water intake(obtained from questionnaires), underlying diseases, serum creatinine, electrolyte, and urine pH. The results of pathogen culture and drug sensitivity test analysis were recorded.

### Sample Collection and Bacteria culture

The isolation and culture of pathogenic bacteria were carried out in strict accordance with the requirements of The National Clinical Test Regulation of Operation (4th edition) [6]. Before stent removal, clean intermittent catheterization was performed, followed by the collection of 10 ml of urine using two disposable sterile syringes. The urine was then placed in two sterile bottles and sent to the microbiology room for bacterial identification and inoculated in blood agar medium and Congo red medium, respectively. The double J stent was removed under sterile operations using a cystoscope and washed repeatedly with saline. After removal of the stent, 3 cm of the tip located in the bladder was sectioned and placed in inoculation bottles with 20 ml of saline and eluted for 1 min with a vortex shaker at 3000 R/min. The broth was used to the multiply the culture on rinsed double J stents inoculated on the Congo red medium. All samples were incubated at 37 ℃ for 24~48h and observed for colony growth and characteristics.

### Bacterial identification and drug sensitivity test

Biofilm-positive bacteria strains were those with black colonies and dry crystals in Congo red medium, on the contrary, pathogenic bacteria strains are colored red [7]. The identification of bacteria and drug sensitivity for pathogenic and biofilm bacteria were performed using a fully automated Vitek 2 compact instrument from Mérieux, France, and the paper diffusion method was performed in strict accordance with the American Clinical Laboratory Standardization Institute(2017 edition) [8]. The medium and drug sensitivity paper are provided by Oxoid Company, UK.

### Statistical analysis

Statistical software SPSS 26.0 was used for statistical analysis. Measurement data consistent with normal distribution were expressed as (‾X ± s). An independent sample T-test was used for comparison between groups. The enumeration data were expressed by frequency or ratio, the total cases no less than 40 and the minimum theoretical frequency over 5 were analyzed by the Chi-square non-correction method, and the minimum theoretical frequency less than 5 and equal to or over 1 were analyzed by the Chi-square correction method. The total cases less than 40 or the minimum theoretical frequency less than 1 were analyzed by Fisher exact probability. The Chi-square test was used for the univariate risk factor analysis and logistic regression was used for the multivariate risk factor analysis. P < 0.05 revealed that the difference was statistically significant.

## Results

### Baseline data of patients

Of these 400 patients, there were 186 males (46.5%) and 214 females (53.5%) with an average age of 55.45 ± 15.80 years (range 18 to 76 years), with hypertension of 98 cases(24.5%), with diabetes of 89 cases(22.2%), with tumor of 40 cases(10%), and with renal dysfunction of 116 cases (29%).(Table 1).

**Table 1:** Baseline Data of Patients

Items	n	Raito(%)
Age		
≤ 60 year	237	59.3
>60 year	163	40.7
Gender		
Male	186	46.5
Female	214	53.5
Diameter of stone		
>1 cm	118	29.5
≤ 1 cm	282	70.5
The Intubation time of the double J stent		
≤ 30 days	297	74.2
>30 days	103	25.8
Urine PH		
≤ 7	376	94.0
>7	24	6.0
With hypertension		
NO	302	75.5
YES	98	24.5
With diabetes		
NO	311	77.7
YES	89	22.3
With cancers		
NO	360	90.0
YES	40	10.0
With hypoalbuminemia		
NO	195	48.7
YES	205	51.3
Renal function		
Normal	284	71.0
Abnormal	116	39.0
Daily water intake		
≥ 2000 ml	232	58.0
<2000 ml	168	42.0

### Isolation of bacteria

A total of 13 strains (3.2%) of biofilm bacteria were detected from urine samples and 168 strains (42%) from double J stent samples (P < 0.05), 95 strains (23.7%) of pathogenic bacteria were separated from urine samples and 117 strains (29.2%) from double J stent samples (P > 0.05). (Table 2)

**Table 2:** Isolation of pathogenic bacteria

sample	Pathogenic Bacteria Culture		Biofilm Bacteria Culture	
	positive	negative	positive	negative
**Double J-Stent**	117(29.2%)	283(70.8%)	168(42.0%)	232(58.0%)
**Urine**	95(23.7%)	305(76.3%)	13(3.2%)	387(96.8%)
X^2^	3.106		171.54	
P	0.07		<0.001	

### Distribution of bacteria

Among the pathogenic bacteria detected in urine samples, 58 strains (61.0%) were Gram-negative bacilli and 37 strains (39.0%) were Gram-positive cocci, *Escherichia coli* (18.9%), *Pseudomonas aeruginosa* (12.6%), *Enterococcus faecalis* (12.6%) and *Enterococcus faecium* (11.6%) were the most common. Among the biofilm bacteria detected in double J stent samples, 106 strains (63.1%) were Gram-negative bacilli and 62 strains (36.9%) were Gram-positive cocci. *Escherichia coli* (19.1%), *Klebsiella pneumoniae* (12.5%), *Pseudomonas aeruginosa* (11.3%) and *Enterococcus faecalis* (9.5%) were the most common. Among the pathogenic bacteria detected in double J stent samples, 71 strains (60.1%) were Gram-negative bacilli and 46 strains (39.3%) were Gram-positive cocci. There were no significant difference in the distribution of bacteria separated from double J stent biofilm bacteria versus urine-cultured pathogens( P > 0.05). There were no significant differences in the distribution of double J-stent biofilm bacteria versus double J stent pathogenic bacteria( P > 0.05).(Table 3).

**Table 3:** Composition Ratio of Pathogenic Bacteria

bacteria	Double-J stent Biofilm Bacteria		Urine Pathogenic Bacteria		Double-J stent Pathogenic Bacteria		Pa	P_b_
	Strain	Ratio (%)	Strain	Ratio (%)	Strain	Ratio (%)		
Gram-negative bacteria	**106**	**63.1**	**58**	**61.0**	**71**	**60.7**		
*Escherichia coli*	32	19.1	18	18.9	22	18.8		
*Klebsiella pneumoniae*	21	12.5	9	9.5	14	12.0		
*pseudomonas aeruginosa*	19	11.3	12	12.6	12	10.3		
*Acinetobacter baumannii*	14	8.3	6	6.3	9	7.7		
*proteus mirabilis*	11	6.5	8	8.4	8	6.8		
*enterobacter cloacae*	6	3.6	4	4.2	4	3.4		
*Morganella morganii*	3	1.8	2	2.1	2	1.7		
Gram-positive bacteria	**62**	**36.9**	**37**	**39.0**	**46**	**39.3**	**0.742**	**0.680**
*Enterococcus faecalis*	16	9.5	12	12.6	14	12.0		
*Enterococcus Faecium*	14	8.3	11	11.6	12	10.3		
*Staphylococcus aureus*	13	7.7	7	7.4	10	8.5		
*Staphylococcus epidermidis*	9	5.4	4	4.2	6	5.1		
*Staphylococcus haemolyticus*	5	3.0	2	2.1	3	2.6		
*Streptococcus agalactiae*	3	1.8	0	0	1	0.8		
*Streptococcus hemolyticus*	2	1.2	1	1.1	0	0		
Total	**168**	**100**	**95**	**100**	**117**	**100**		

P_a_: Comparison of distribution of double J-stent biofilm bacteria and urine pathogenic bacteria; P_b_: Comparison of distribution of double J-stent biofilm bacteria and double J-stent pathogenic bacteria

### Drug resistance

Among pathogenic bacteria and biofilm bacteria, Gram-negative bacilli were extremely resistant to semisynthetic penicillin (e.g. ampicillin, piperacillin) and were slightly higher resistance for first-, second-, and third-generation cephalosporins(e.g. cefazolin, cefuroxime, ceftriaxone, cefotaxime, cefoxitin, ceftazidime), quinolones(e.g., levofloxacin, ciprofloxacin), tetracycline, cotrimoxazole and nitrofurantoin. However, they were extremely sensitive to drugs with β-lactamase inhibitors, fosfomycin tromethamine and carbapenems. Gram-positive cocci were highly resistant to penicillins, cephalosporins, quinolones, and erythromycin drugs, but were extremely sensitive to vancomycin, linezolid, rifampicin, and tigecycline. Greater drug resistance was observed in biofilm bacteria cultivated with double J stent versus pathogenic bacteria cultivated with urine or double J stent, especially cephalosporins, carbapenems and quinolones (P < 0.05).(Tables 4 and 5).

**Table 4:** Drug Resistance in Gram-Negative Bacteria

Antibacterial Drugs	Double J-Stent Biofilm Bacteria		Urine Pathogenic Bacteria		Double J-Stent Pathogenic Bacteria		Pa	P_b_
	Strain(106)	Ratio(%)	Strain(58)	ratio(%)	Strain(71)	ratio(%)		
Cefoperazone/sulbactam	7	6.6	2	3.4	4	5.6	0.396	0.792
Meropenem	17	16.0	2	3.4	3	4.2	0.016	0.014
Tigecycline	10	9.4	3	5.2	4	5.6	0.334	0.358
Ertapenem	20	18.8	4	6.8	4	5.6	0.038	0.011
Fosfomycin tromethamine	17	16.0	7	12.6	8	11.2	0.491	0.371
Imipenem	22	20.7	6	10.3	7	9.8	0.090	0.054
Amikacin	27	25.5	9	15.5	12	16.9	0.140	0.177
Ticacillin - clavulanic acid	25	24.5	10	17.2	13	18.3	0.343	0.402
Piperacillin tazobactam	30	28.3	9	15.5	11	15.4	0.065	0.047
Tobramycin	38	35.8	12	20.7	14	19.7	0.004	0.001
Gentamicin	33	31.1	14	24.1	18	25.3	0.343	0.405
Levofloxacin	56	52.8	17	29.3	20	28.1	0.003	0.001
Ciprofloxacin	66	62.2	21	36.2	26	36.6	0.001	<0.001
Cefepime	33	31.1	16	27.5	19	26.7	0.635	0.531
Ceftazidime	62	58.5	19	32.7	22	30.9	0.001	<0.001
Amoxicillin/clavulanic acid	45	42.4	21	36.2	25	35.2	0.435	0.334
Cefufoxin	68	64.1	23	39.6	28	39.4	0.002	0.001
Cefoxitin	48	45.2	20	34.5	29	40.8	0.179	0.559
Aztreonam	52	49.0	25	43.1	31	43.6	0.465	0.480
Ceftriaxone	72	67.9	26	44.8	30	42.2	0.003	<0.001
Cefotaxime	57	53.7	21	36.2	24	33.8	0.031	0.010
Tetracycline	60	56.6	28	48.2	35	49.2	0.306	0.339
Cotrimoxazole	62	58.5	27	46.5	34	47.8	0.142	0.165
Nitrofurantoin	45	42.4	13	22.4	18	25.3	0.010	0.019
Cefazolin	88	83.0	23	55.2	45	63.3	<0.001	0.003
Piperacillin	90	84.9	45	77.6	56	78.8	0.240	0.300
Ampicillin	99	93.3	52	89.6	60	84.5	0.396	0.055

P_a_: Comparison of drug resistance of double J-stent biofilm bacteria and urine pathogenic bacteria; P_b_: Comparison of drug resistance of double J-stent biofilm bacteria and double J-stent pathogenic bacteria

**Table 5:** Drug Resistance of Gram-Positive Bacteria

Antibacterial Drugs	Double J-Stent Biofilm Bacteria		Urine Pathogenic Bacteria		Double J-Stent Pathogenic Bacteria		Pa	P_b_
	Strain(62)	Ratio(%)	Strain(37)	ratio(%)	Strain(46)	ratio(%)		
Linezolid	6	9.7	0	0	0	0	0.050	0.029
Vancomycin	1	1.6	0	0	1	2.1	0.437	0.830
Tigecycline	2	3.2	0	0	0	0	0.269	0.218
Rifampicin	10	16.1	4	10.8	6	13.0	0.462	0.655
Nitrofurantoin	19	30.6	6	16.2	8	17.4	0.109	0.115
Cotrimoxazole	18	29.0	8	21.6	11	23.9	0.417	0.552
Moxifloxacin	30	48.4	10	27.0	12	26.0	0.036	0.018
Quinopltin/dafopltin	20	32.2	11	29.7	14	30.4	0.792	0.839
Levofloxacin	40	64.5	13	35.1	14	30.4	0.004	<0.001
Tetracycline	33	53.2	17	45.9	19	41.3	0.483	0.220
Ciprofloxacin	34	54.8	18	48.6	22	47.8	0.550	0.470
HGEN	19	30.6	9	24.3	12	26.0	0.499	0.604
Gentamicin	30	48.3	18	48.6	20	43.3	0.974	0.612
HLSR	13	20.9	7	18.9	11	23.9	0.806	0.715
Ampicillin	50	80.6	20	54.0	24	52.1	0.004	0.001
Penicillin G	51	82.2	22	59.4	25	54.3	0.012	0.001
Ampicillin/sulbactam	40	64.5	20	54.0	23	50.0	0.302	0.130
Amoxicillin/clavulanic acid	39	62.9	22	59.4	24	52.1	0.733	0.263
Oxacillin	47	75.8	21	56.7	23	50.0	0.048	0.005
Ceftriaxone	46	74.2	20	54.0	23	50.0	0.039	0.009

P_a_: Comparison of drug resistance of double J-stent biofilm bacteria and urine pathogenic bacteria; P_b_: Comparison of drug resistance of double J-stent biofilm bacteria and double J-stent pathogenic bacteria

### Analysis of the influencing factors

Univariate analysis showed that patients with age more than 60 years, catheterization time more than 30 days, urinary PH greater than 7, daily water intake less than 2000 milliliters, hypoproteinemia, renal dysfunction, hypertension, diabetes and tumor were more susceptible to biofilm bacterial infections. These statistically significant variables were then included in multivariate logistic regression analysis, and the results showed that advanced age, long-term catheterization, insufficient water intake, hypoproteinemia, abnormal renal function, and diabetes were independent risk factors for biofilm bacteria infection associated with double J stent(P < 0.05).(Tables 6 and 7).

**Table 6:** Univariate analysis of risk factors

Items	Biofilm Bacteria Culture			X^2^	P
	Negative	Positive			
Age					
≤ 60 year	164		73	29.940	<0.001
>60 year	68		95		
The Intubation time of the double J-stent					
≤ 30 days	162		135	5.650	0.017
>30 days	70		33		
Urine PH					
≤ 7	213		163	4.696	0.030
>7	19		5		
With hypertension					
NO	186		116	6.519	0.011
YES	46		52		
With diabetes					
NO	198		113	18.418	<0.001
YES	34		55		
With cancers					
NO	215		145	4.383	0.036
YES	17		23		
With hypoalbuminemia					
NO	138		57	25.468	<0.001
YES	94		111		
Renal function					
Normal	191		93	34.424	<0.001
Abnormal	41		75		
Daily water intake					
≥ 2000 ml	99		133	23.373	<0.001
<2000 ml	33		135		

**Table 7:** Logistic regression analysis of risk factors

Risk factors	B	Wald	OR	P	95%CI	
					Min	Max
Advanced Age	0.695	8.153	1.923	0.009	1.177	3.142
The Intubation time of the double J stent	3.129	8.453	22.844	0.004	2.777	188.258
With diabetes	0.997	9.231	2.709	0.002	1.424	5.154
With hypoalbuminemia	0.930	10.715	2.533	0.001	1.452	4.420
Renal function	0.909	10.586	2.483	0.001	1.436	4.294
Daily water intake	3.776	12.741	43.649	<0.001	5.489	347.102

## Discussion

With the extension of indwelling time, double J stent associated urinary tract infections would inevitably occur and become a common complication. As early as 1676, Antonie observed the existence of bacterial biofilms from dental plaque, which exist widely in the natural environment. It was not until 1978 that Costerton J and colleagues [9] first put forward theories related to biofilms. Bacterial biofilms can be formed on the surface of various implants and internal mucosa and induce the aggravation of drug resistance through a variety of mechanisms. Catheter-related infections play a key role in clinical chronic infections.

This study confirmed that the detection rate of biofilm bacteria in the double J stent was significantly higher than that in urine, which may be due to the lack of normal protective mechanisms of body tissue on the surface of the double J stent. Furthermore, Previous research indicated that salts and other substances in urine were deposited on the double J stent within minutes of implantation of the double J stent, creating favorable conditions for bacterial colonization. Common pathogens of urinary tract infections, such as *Escherichia coli*, *Proteus*, *Staphylococcus* and *Enterococcus*, can colonize the surface of ureteral stents within 24 h [10], and fibrin membrane deposition appears on the catheter within 24 to 48 h after catheter implantation, which become a scaffold for bacterial migration adhesion [11]. The process of biofilm formation includes several parts: bacterial adhesion to the surface of the implant; formation of microcolonies; maturation of biofilm and dissemination of biofilms.

There were no significant differences in the distribution of bacteria separated from double J stent biofilm bacteria versus urine-cultured pathogens( P > 0.05). There were no significant differences in the distribution of double J stent biofilm bacteria versus double J-stent pathogenic bacteria. Our research demonstrated that the detection rate of Gram-negative bacteria was high; *Escherichia coli* were the most common(18.9% / 19.1% / 18.8%), followed by *Pseudomonas aeruginosa*, *Klebsiella pneumoniae*, and *Proteus mirabilis.* Furthermore, *Enterococcus* and *Staphylococcus* accounted for a high proportion of Gram-positive bacteria, which was highly consistent with the common pathogenic bacteria species of the urinary system and was basically in line with the previous research results [7, 12]. *Escherichia coli* was the most common pathogen, which may be related to the adhesins distributed at the apex of the E.coli cilia, which could combine with specific receptors on the surface of muco-associated cells and thus remained in the urinary tract for a long time [13]. Undoubtedly, this deserves our vigilance.

The analysis of drug resistance of pathogenic bacteria in our study confirmed that Gram-negative bacilli were extremely resistant to semisynthetic penicillin and were slightly higher resistance for first, second and third generation cephalosporins, quinolones, tetracycline, cotrimoxazole, and nitrofurantine. On the contrary, they were extremely sensitive to drugs with β-lactamase inhibitors, fosfomycin tromethamine, and carbapenems, which was basically in line with the previous research results [7, 12]. Gram-positive cocci were highly resistant to penicillins, cephalosporins, quinolones, and erythromycin drugs, which own to their unique natural resistance mechanisms. It is noteworthy that greater drug resistance was observed in biofilm bacteria versus pathogenic bacteria cultivated with urine or double J stent (P < 0.05). The exacerbation of drug resistance of biofilm bacteria may be related to the following factors:①Biofilm plays a barrier role and shows a cluster distribution structure on the whole with a certain gap between the two clusters [14], which can protect the bacteria inside the organism from antibiotics and the body’s immune system. ②Some components of the biofilm can alter the permeability of antimicrobial agents. ③The structure of the biofilm is heterogeneous with concentration gradients of nutrients and signal molecules in the internal environment. RATH and his workers [15] found that there were always inactive biomolecules at the bottom of the biofilm, which could escape the killing effect of antimicrobial agents by changing into a spore-like state and reducing their metabolism and growth rate.④The quorum-sensing system, anti-immune clearance mechanism, special growth characteristics, and the opening of biofilm resistance genes [16] also play an major role in the aggravation of drug resistance. The bacteriological biofilm consists mainly of glycocalyx, extracellular DNA(eDNA), lipids, and proteins. Imipenem can play an anti-biofilm role by reducing the eDNA component of the biofilm [17]. However, β-lactamase is also presented in ground substance of biofilm [18], which may lead to a lower susceptibility of imipenem or meropenem to carbapenem-resistant strains. Most pathogenic bacteria separated from the double J stent were floating bacteria, whose biological characteristics were similar to those of urine pathogens. Unlike biofilm bacteria, they have not yet developed complex resistance mechanisms. This may explain the differences in drug resistance between double J stent biofilm bacteria and double J stent pathogenic bacteria. Through these resistance pathways, biofilm bacteria can survive in antibiotics with concentrations of 1,000 to 1,500 times higher than those in which floating bacteria can be eliminated, causing great difficulties in clinical treatment.

The more severe biofilm bacteria infections, the more attention of risk factors need to be paid. Our analysis further showed that patients with advanced age, inadequate water intake, long-term catheterization, or accompanied by underlying diseases had a higher incidence of biofilm bacteria associated infections. Scholars have found that bacterial biofilm formation is positively correlated with catheter implantation time, immune dysfunction, diabetes, etc., and serum creatinine greater than 176µmol/L and long operation time are risk factors [19], which is similar to our research results. Degeneration of organ function in the elderly, chronic malnutrition, and decreased number and function of immune cells in people with hypoproteinemia, insufficient urine volume due to chronic inadequate water intake, and the indiscriminate use of antibiotics, all factors provide conditions for bacterial propagation. Previous studies have confirmed that a high glucose environment can promote the formation of staphylococcal biofilm [20]. In addition, hydronephrosis and acute kidney injury caused by obstruction of upper urinary tract can also provide conditions for biofilm bacteria associated infections by producing a large number of metabolites and lipid peroxidation. Relevant evidence also demonstrated that hydronephrosis and acute renal insufficiency can cause urinary tract infection in patients with upper urinary calculi [21]. Studies revealed that a longer indwelling time of stents was associated with a higher bacteria detection rate [7, 22]. Bacteria are easy to attach to long-term indwelling stent, multiply and produce biofilms. With the extension of the indwelling time of the stent, encrustation formed on the surface of the stent. Encrustation will block stents and the ureter, eventually promoting the formation of biofilm bacteria and leading to sepsis. We believe that the prevention of biofilm bacteria associated infections should be carried out in various ways: ①Controlling risk factors of infection in susceptible persons, such as treating underlying diseases, reducing catheterization time, increasing the amount of drinking water, and rationally using antibiotics.②Using antimicrobial materials(coating biomaterials with antibiotics or silver ions) in the hope of meeting the optimal local concentration to prevent bacterial adhesion, which has been shown to be feasible [23, 24].

There are still some limitations in this study. As a single-center study, the source and selection of the research objects affect the results to some extent. There may be irregularities in the collection and treatment of the samples, which may affect the results of pathogen culture. The sample size of some indexes is small, which may cause an experimental error. We believe that large sample, multi-center studies need to be further developed.

## Conclusion

Taken together, the positive rate and drug resistance of biofilm bacteria separated from double J stent were significantly higher than pathogenic bacteria in urine, which deserves the concern of medical workers. Furthermore, more attention should be paid to the factors that influence biofilm bacteria infections.

## Data Availability

All relevant data and materials are included in this article.

## Declarations

### Accordance to relevant guidelines

All methods were carried out in accordance with relevant guidelines and regulations in the Declaration of Helsinki under the heading ‘Ethics and consent to participate’.
